# Right Indirect Carotid-Cavernous Fistula Presenting With Isolated Oculomotor Palsy: A Diagnostic Challenge and Successful Endovascular Management

**DOI:** 10.7759/cureus.93989

**Published:** 2025-10-06

**Authors:** Sujith Jayaprakash, Jithin Bose, Gigy V Kuruttukulam, Senthil Kumar

**Affiliations:** 1 Neurology, Rajagiri Hospital, Aluva, IND; 2 Medicine, Government Medical College, Thiruvananthapuram, Thiruvananthapuram, IND

**Keywords:** 3rd nerve palsy, carotid-cavernous sinus fistula, digital subtraction angiography(dsa), endovascular coil embolisation, indirect carotid cavernous fistula, tolosa-hunt syndrome

## Abstract

Carotid-cavernous fistulas are abnormal communications between the carotid arterial system and the cavernous sinus. Indirect carotid-cavernous fistulas are low-flow lesions that may sometimes present subtly and mimic inflammatory or neoplastic cavernous sinus disease. We report the case of a 76-year-old man with diabetes and hypertension who developed painless diplopia and right-sided partial ptosis over two weeks. Clinical examination revealed right incomplete third nerve palsy with pupillary involvement. Magnetic resonance imaging showed enhancing soft tissue thickening in the right cavernous sinus, raising suspicion of an inflammatory process. He was treated with corticosteroids after appropriate glycemic control but showed no improvement. Further evaluation with positron emission tomography and cerebrospinal fluid analysis excluded malignancy and infection, although serum galactomannan and IgG4 were mildly elevated, complicating the diagnostic picture. The lack of response to steroids and inconclusive imaging prompted consideration of a vascular etiology. Digital subtraction angiography ultimately confirmed an indirect carotid-cavernous fistula draining via the inferior petrosal sinus. The patient underwent transvenous coil embolization under general anesthesia, achieving near-complete occlusion of the fistula. The procedure was uneventful, and the patient experienced complete resolution of diplopia and ptosis at one-month follow-up. He was discharged in stable condition with multidisciplinary follow-up. This case highlights how indirect carotid-cavernous fistulas can mimic other cavernous sinus pathologies, how misleading ancillary tests may complicate diagnosis, and the critical importance of angiographic evaluation when non-invasive imaging is inconclusive. Endovascular transvenous coil embolization provided safe and effective treatment with favorable clinical outcomes.

## Introduction

Carotid-cavernous fistulas (CCFs) are abnormal shunts between the carotid artery system and the cavernous sinus. The Barrow classification divides them into direct and indirect (dural) types [1]. Indirect CCFs are low-flow lesions that often present insidiously. Classic findings include chemosis, proptosis, and orbital bruit. However, posteriorly draining fistulas may present solely with cranial nerve palsies, particularly involving the oculomotor nerve, which makes diagnosis challenging. Although isolated third nerve palsy has several possible causes, including vascular, compressive, and inflammatory lesions, in the context of cavernous sinus pathology, an indirect carotid-cavernous fistula should always be considered among the leading differentials. Within the cavernous sinus and orbital apex, lesions may arise from neoplasm, thrombosis, inflammation, vascular fistulas, or infiltrative disorders [2]. Thus, patients presenting with isolated ocular motor deficits require careful consideration of vascular, neoplastic, inflammatory, and infectious etiologies.

In our patient, several diagnostic pitfalls complicated evaluation. The presence of diabetes initially led to suspicion of microvascular ischemic third nerve palsy; however, the pupillary involvement and a hemoglobin A1C (HbA1C) of 7% made this less likely and suggested a compressive or vascular process [3]. MRI findings raised concern for Tolosa-Hunt syndrome, prompting corticosteroid therapy, but the lack of clinical response required reconsideration [4]. A mildly positive galactomannan and elevated IgG4 further confounded the picture, leading to empirical antifungal treatment despite a weak correlation [5]. These overlapping impressions delayed recognition of the underlying indirect carotid-cavernous fistula.

Indirect CCFs themselves can develop spontaneously or be associated with predisposing conditions. Reported risk factors include trauma, pregnancy, sinusitis, cavernous sinus thrombosis, hypertension, and connective tissue disorders. Because of their variable clinical manifestations, these fistulas can mimic other cavernous sinus pathologies. Magnetic resonance imaging (MRI)/magnetic resonance angiography (MRA) and CT angiography can suggest the diagnosis, but digital subtraction angiography remains the gold standard for confirmation [6]. In this report, we present a case of indirect CCF in an elderly man who presented with isolated oculomotor palsy, to highlight the importance of considering vascular lesions alongside other differential diagnoses and common clinical pitfalls in the cavernous sinus region [7].

## Case presentation

A 76-year-old male, known hypertensive and diabetic for more than two decades, presented with a two-week history of progressive diplopia and drooping of the right upper eyelid. The diplopia was binocular and predominantly horizontal, more noticeable on left gaze, consistent with weakness of the right medial rectus due to partial third nerve palsy [8]. There was no history of headache, orbital pain, periorbital swelling, proptosis, or redness. He denied fever, weight loss, or constitutional symptoms. There was no prior ocular surgery or trauma.

On ocular examination, the right eyelid showed ptosis obscuring half of the pupil, and there was restriction of adduction and elevation of the right eye. Depression and abduction were preserved, likely due to intact superior oblique and lateral rectus function. The right pupil was sluggishly reactive to light and slightly larger than the left, consistent with partial oculomotor nerve palsy, but there was no relative afferent pupillary defect (RAPD), excluding significant optic nerve involvement. Visual acuity at presentation was 6/18 in the right eye and 6/9 in the left eye, and the mild right-sided reduction was attributed to diplopia and pupillary involvement rather than optic nerve dysfunction [9]. The remainder of the cranial nerves were intact, and motor, sensory, and cerebellar examination showed no abnormalities. Limb strength and reflexes were preserved.

**Figure 1:**
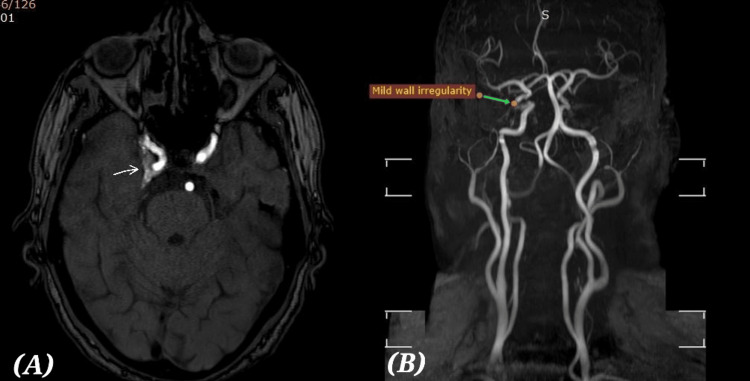
MRI + Angiogram (A) MRI (axial time of flight): Asymmetric T2 intermediate signal intensity enhancing soft tissue in the region of right cavernous sinus causing bulging of the right lateral margin and partial obliteration of the superior aspect of right Meckel's cave suspicious of Tolosa-Hunt (B) MRA: Mild wall irregularities of the right internal carotid artery (ICA) cavernous segment likely due to the MRI findings

MRI of the brain and orbits demonstrated asymmetric T2 intermediate signal intensity enhancing soft tissue in the region of right cavernous sinus causing bulging of the right lateral margin and partial obliteration of the superior aspect of right Meckel’s cave. These findings raised the possibility of an inflammatory process such as Tolosa-Hunt syndrome (Figure 1A, 1B). On this basis, oral corticosteroid therapy was initiated following glycaemic optimization [10]. However, after one week, there was no appreciable clinical improvement in diplopia or ptosis, prompting reconsideration of the working diagnosis.

Further investigations added diagnostic complexity. Positron emission tomography (PET)-CT showed no fluorodeoxyglucose (FDG)-avid lesion, reducing the likelihood of neoplasm [11]. CSF analysis was unremarkable, excluding central nervous system infection or malignant infiltration. Serum galactomannan was mildly positive (0.65), and IgG4 levels were elevated (235 mg/dL). ENT consultation recommended empirical antifungal therapy, and the patient was started on oral voriconazole. Despite this, the clinical picture remained incongruous. The raised IgG4 suggested possible IgG4-related disease, though the absence of systemic involvement or histopathology limited certainty [12].

**Figure 2:**
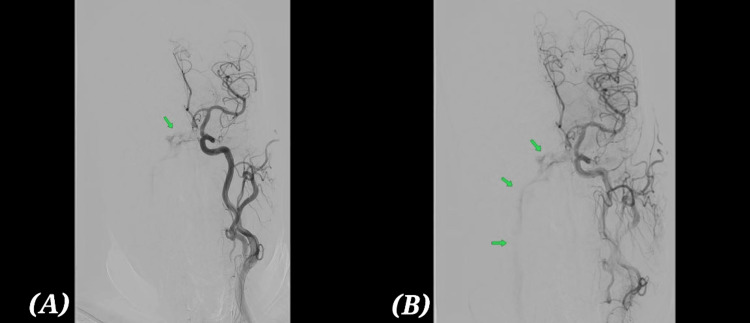
Digital subtraction angiography (DSA) before embolization (A) Starting of venous filling and (B) remaining venous filling in the arterial phase showing indirect (Barrow type D) carotid-cavernous fistula. Arrow mark indicate venous filling.

Given persistent ocular deficits, lack of steroid response, and inconclusive systemic workup, a vascular etiology was revisited. Digital subtraction angiography (DSA) revealed an indirect CCF with venous drainage through the inferior petrosal sinus (Figure 2A, 2B). This explained the progressive cranial neuropathy in the absence of orbital congestion [13].

**Figure 3:**
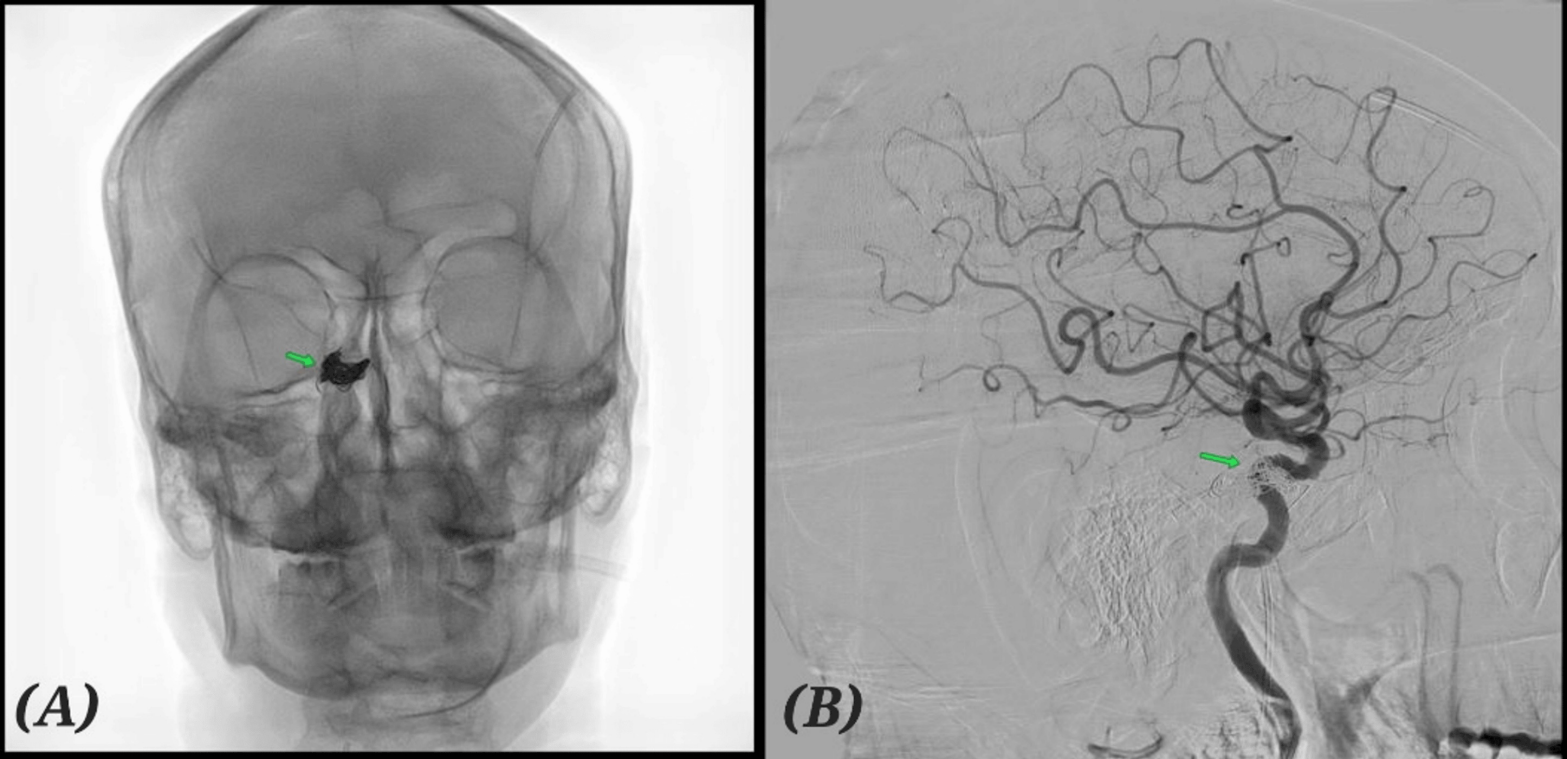
Digital subtraction angiography (DSA) status post venous coil embolization (A) AP (anteroposterior) projection. (B) Lateral projection. Arrow mark indicates coil insitu. No arteriovenous shunting.

The patient underwent transvenous embolisation of the cavernous sinus via the inferior petrosal sinus approach [14]. Multiple detachable coils were deployed, achieving near-complete occlusion of the fistula (Figure 3A, 3B). The procedure was uneventful.

**Figure 4:**
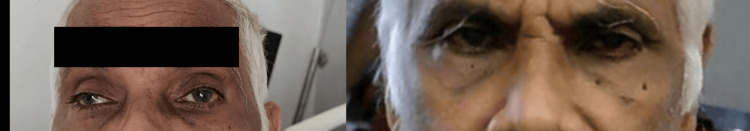
Pre- and post-embolization clinical pictures (A) Pre-embolization picture showing ocular features of third nerve palsy. (B) Post-embolization picture showing complete recovery.

Ocular examination demonstrated striking improvement. Before embolisation, the right eye was positioned slightly downward and outward, consistent with partial third nerve palsy, and mild ptosis was evident (Figure 4A). After embolisation, there was complete resolution of ptosis and realignment of the visual axes, with resolution of diplopia (Figure 4B).

Remarkably, the neurological recovery was much faster than typically expected. Within a very short time following embolisation, the patient’s ptosis and ocular motility deficits resolved almost completely. This unusually rapid improvement, especially in a case of cranial nerve palsy secondary to an indirect CCF, was both striking and clinically surprising [15].

The patient was discharged in stable condition on day three post-procedure. At one-month follow-up, he remained symptom-free, with full ocular motility, normal visual acuity, and no recurrence of cranial nerve dysfunction. He continues under multidisciplinary follow-up.

## Discussion

This case illustrates a diagnostic pitfall of indirect CCF. Unlike direct fistulas, which often present with dramatic ocular congestion, indirect CCFs may present subtly, sometimes only with cranial nerve palsies [6]. Our patient’s MRI suggested an inflammatory cavernous sinus process, leading to an initial misdiagnosis of Tolosa-Hunt syndrome. Serum galactomannan and IgG4 levels were obtained to evaluate for possible fungal infection and IgG4-related inflammatory disease, respectively, based on the imaging findings and lack of response to corticosteroids. The elevations in these parameters added to the diagnostic uncertainty. These factors underscore how infectious and autoimmune markers can confound the diagnosis of vascular lesions in the cavernous sinus.

DSA remains the gold standard for diagnosing CCF, particularly when MRI/MRA findings are nonspecific. The presence of posterior drainage patterns often explains the absence of orbital congestion. In this case, transvenous coil embolisation was chosen and resulted in excellent angiographic and clinical outcomes. Endovascular management remains first-line therapy, with high success rates and low complication risk [16].

The isolated third nerve palsy in our patient can be explained by the hemodynamic pattern of the fistula. Indirect CCFs that drain posteriorly into the inferior petrosal sinus elevate venous pressure within the cavernous sinus without producing the orbital venous congestion typically seen with anterior drainage. This localized venous hypertension can compress or ischemically injure the cranial nerves traversing the cavernous sinus, most commonly the oculomotor nerve, leading to isolated third nerve palsy without proptosis, chemosis, or bruit. The importance of considering vascular, infectious, and inflammatory conditions in the differential diagnosis of isolated cranial neuropathy cannot be overstated [17].

Clinicians should maintain a high index of suspicion for CCF in patients with isolated cranial neuropathies and cavernous sinus abnormalities on MRI, especially when initial therapy for alternative diagnoses (e.g., steroids for presumed Tolosa-Hunt) fails. This case highlights the importance of considering vascular causes even in the presence of misleading serological or imaging findings.

## Conclusions

Indirect CCF should be considered in patients presenting with isolated ocular motor palsies and subtle cavernous sinus abnormalities on imaging. When noninvasive studies are inconclusive or when patients fail to respond to initial medical therapy, DSA remains essential for definitive diagnosis. Clinicians should suspect indirect CCF in cases of atypical or isolated cranial nerve palsies, particularly when associated with pupillary involvement, and proceed to angiography when inflammatory or infectious causes have been excluded. Endovascular transvenous coil embolisation offers safe and effective management, preventing progression of complications and providing the potential for reversal of cranial neuropathies, as demonstrated in this case.
